# Measuring rhythms of vocal interactions: a proof of principle in harbour seal pups

**DOI:** 10.1098/rstb.2021.0477

**Published:** 2023-04-24

**Authors:** Marianna Anichini, Koen de Reus, Taylor A. Hersh, Daria Valente, Anna Salazar-Casals, Caroline Berry, Peter E. Keller, Andrea Ravignani

**Affiliations:** ^1^ Comparative Bioacoustics Group, Max Planck Institute for Psycholinguistics, 6525 XD Nijmegen, The Netherlands; ^2^ Department of Biological Sciences, Faculty of Natural Sciences, Norwegian University of Science and Technology N-6025 Ålesund, Norway; ^3^ Hanse-Wissenschaftskolleg Institute for Advanced Study, 'Brain' Research Area, 27753 Delmenhorst, Germany; ^4^ Division Animal Physiology and Behaviour, Department for Neuroscience, Carl von Ossietzky University Oldenburg, 26129 Oldenburg, Germany; ^5^ Artificial Intelligence Laboratory, Vrije Universiteit Brussel, 1050 Brussels, Belgium; ^6^ Donders Institute for Brain, Cognition and Behaviour, Radboud University, 6500 GL Nijmegen, The Netherlands; ^7^ Department of Life Sciences and Systems Biology, University of Turin, 10123 Turin, Italy; ^8^ Research Department, Sealcentre Pieterburen, 9968 AG Pieterburen, The Netherlands; ^9^ MARCS Institute for Brain, Behaviour and Development, Western Sydney University, Penrith, NSW 2751, Australia; ^10^ Center for Music in the Brain, Department of Clinical Medicine, Aarhus University and The Royal Academy of Music Aarhus/Aalborg, 8000 Aarhus, Denmark

**Keywords:** behavioural interaction, asynchrony, circular statistics, categorical rhythms, time-series analysis, interactive vocal rhythm

## Abstract

Rhythmic patterns in interactive contexts characterize human behaviours such as conversational turn-taking. These timed patterns are also present in other animals, and often described as rhythm. Understanding fine-grained temporal adjustments in interaction requires complementary quantitative methodologies. Here, we showcase how vocal interactive rhythmicity in a non-human animal can be quantified using a multi-method approach. We record vocal interactions in harbour seal pups (*Phoca vitulina*) under controlled conditions. We analyse these data by combining analytical approaches, namely categorical rhythm analysis, circular statistics and time series analyses. We test whether pups' vocal rhythmicity varies across behavioural contexts depending on the absence or presence of a calling partner. Four research questions illustrate which analytical approaches are complementary versus orthogonal. For our data, circular statistics and categorical rhythms suggest that a calling partner affects a pup's call timing. Granger causality suggests that pups predictively adjust their call timing when interacting with a real partner. Lastly, the ADaptation and Anticipation Model estimates statistical parameters for a potential mechanism of temporal adaptation and anticipation. Our analytical complementary approach constitutes a proof of concept; it shows feasibility in applying typically unrelated techniques to seals to quantify vocal rhythmic interactivity across behavioural contexts.

This article is part of a discussion meeting issue ‘Face2face: advancing the science of social interaction’.

## 1. Introduction and methodological approaches

### (a) Rhythmic interaction and turn-taking

Humans adopt precise signalling behaviours to exchange information [1,2]. No matter the signal modality (e.g. acoustic and visual), an interactive event between sender and receiver is governed by a timed structure [3–6]. The structured exchange of communicative turns (i.e. turn-taking) characterizes our capacity for social interaction, enabling us to communicate with others [7,8]. The study of interactive rhythms—how two (or more) individuals coordinate their signalling in time—is an emerging field of research, with more recent work extending structural analyses of communication signals, once restricted to human spoken conversation, to other species [3,9]. Turn-taking in communication has been documented in primates [10] and in other non-primate species [6,11–13]. For example, call exchanges in adult common marmoset monkeys (*Callithrix jacchus*) exhibit coupled oscillator dynamics, like those observed in human turn-taking [14]. Two key components of turn-taking are the flexible organization and distribution of turns, and the temporal relationship between adjacent turns [11]. In humans, face-to-face interactions require participants to be, among others, both socially and temporally sensitive [8]. Does behavioural context also affect signal timing in non-human animals? Comparative investigations on interactive vocal timing in mammals may help uncover shared turn-taking mechanisms, potentially providing more insights into their evolution.

### (b) Methodological approaches and challenges for studying rhythmic interaction

Expanding the human turn-taking framework to other species is currently hindered by, among other things, the lack of suitable methodological approaches [11,15]. Cross-species frameworks exist [16], but open questions still remain in animal face-to-face interaction, such as: which analytical methodologies used to investigate turn-taking in humans may reveal temporal adjustments in other species? Can turn-taking arise from non-cooperative behavioural interactions? Can methods developed for individual rhythm analyses be used to study rhythmic interaction? Can parametric models for human rhythmic prediction and reaction detect similar features in other species? These open questions require suitable animal models and quantitative methods.

Different forms of vocal rhythmic interaction, such as synchronous chorusing and turn-taking, have been mainly studied in mammals within a cooperative dynamic, like parent–infant and male–female dyads. Time-series analyses like Granger causality have shown temporal interdependence between vocalizations in male–female pairs [17,18] and movements [19] of non-human primates. Circular statistics is another method to study timing adjustments in interactions and has been used in previous animal work, including a seal pup playback experiment [20] and a study on parent–infant monkey interaction [21]. Categorical rhythms—those for which the temporal intervals between signal onsets are distributed categorically rather than uniformly—are a universal characteristic of human music, which is often produced in an interactive context [22]. Similar rhythms are also present in non-human animal songs (e.g. thrush nightingales [22], indris [23]), but whether such rhythms characterize other interactive non-song vocalizations, such as animal calls, is unknown [22,23]. Roeske *et al*. [22] hypothesized that categorical rhythms play a role in calls produced to attract and hold conspecific attention, by making sequences of vocalizations more predictable to listeners. Categorical rhythm analyses could therefore be an interesting method to test the predictability of vocal sequences in non-human animal interactions. Lastly, the ADaptation and Anticipation Model (ADAM), originally developed to model the mechanisms for interpersonal coordination in humans [24], has been adopted to probe sensorimotor and cognitive mechanisms underlying temporal dynamics in interaction [25,26]. Although ADAM is designed for ‘simultaneous chorusing’, it could also be used for a mixture of bouts of synchrony, turn-taking and other regimes [20,27].

In this proof of concept study, we showcase how these methodological tools—Granger causality, circular statistics, categorical rhythm analysis and ADAM—can be applied to a new animal model: the harbour seal (*Phoca vitulina*). To illustrate the use and compatibility of these different analytical methods, we show how they can be used to better understand the rhythmic communication of a small sample of harbour seal pups in different behavioural contexts.

### (c) Our animal model

The ‘vocal learning-beat perception and synchronization’ (VL-BPS) hypothesis states that only vocal learning species—those capable of producing new vocalizations or modifying existing ones based on auditory experience—may possess advanced rhythmic abilities [28,29]. This hypothesis is inherently cross-modal: it suggests a strong link between audition and timed movement. For example, Snowball, a sulfur-crested cockatoo (*Cacatua galerita eleonora*), was shown to perceive auditory rhythms at different tempi and to predictively synchronize his body movements to them [30]. Parrots are phylogenetically distant from humans and, among mammals, pinnipeds (seals, sea lions and walruses) are one of the vocal learning groups (besides humans, bats, elephants and cetaceans). Pinnipeds may well be the best mammalian model for testing the VL-BPS hypothesis—the ability to extract a beat from periodic acoustic stimuli and entrain to it in a predictive and adaptive manner—since some species show vocal mimicry and plasticity [31,32] and others can keep a beat [33]. These characteristics, parallelling human abilities, make pinnipeds an ideal animal clade for comparative research on the origins of rhythmic communicative behaviour.

Harbour seals exhibit both vocal flexibility [32,34] and rhythmic interactivity [20], and are particularly vocal in the first few weeks of life [35]. During the lactation period, harbour seal pups emit ‘mother attraction calls' (hereafter ‘calls’) to draw their mothers' attention [36]. Mothers are silent and use the individual vocal signatures in these calls to recognize their pups [35,37]. Against the acoustically complex backdrop of large mother–pup rookeries, rhythmically tuned pup calls could constitute a socio-ecologically selected trait that allows individual pups to avoid conspecific call overlap by adjusting the timing of their own call onsets. Such timing plasticity could allow a pup to be more acoustically conspicuous and increase its chances of successful reunions with its mother. Unlike cooperative types of turn-taking (e.g. in humans and in common marmosets [38]) harbour seal pups’ interactions are a by-product of neighbouring pups vocalizing to attract their silent mothers and are thus probably competitive.

To date, only two papers studied vocal rhythms in harbour seals, crucially both focusing on single individuals [20,27]. The first study was a playback experiment in which a pup vocally interacted with sounds broadcasted from a loudspeaker [20]. The pup adjusted the timing of its calls in an asynchronous manner by responding to the broadcasted conspecific calls with a non-uniformly distributed response phase whose mean approximated 90° [20]. The second study looked at the presence and development of vocal rhythms in three harbour seal pups [27]. Complementary analytical approaches showed how the pups' *individual* calling patterns gained more rhythmic structure over time [27]. However, a major limitation of both studies was the lack of sociality (i.e. individuals were tested alone) and, by extension, interactivity (i.e. the stimuli did not adapt to the response of the tested animals).

### (d) Aims and research questions

In this work, we show how vocal interactive rhythmicity in non-human animals can be quantified using a multi-method approach spanning various research domains (e.g. temporal, social and cognitive) (table 1). We illustrate this approach through four research questions, all of which relate back to whether harbour seal vocal interactive rhythmicity varies in different behavioural contexts (table 1). While our sample sizes are too small to enable species-wide inferences, they are sufficient to illustrate how methods typically used to study human communication can be adopted to study interactivity in animal communication. The goal of this paper is thus to outline a quantitative roadmap that future research can follow. Circular statistics and categorical rhythm analysis are used to address the first question about temporal adjustment in interaction: *‘does the presence of a calling partner affect the call timing of individual pups?’* (Q1). The next two questions consider the effect of behavioural context on temporal adjustment: ‘*does the type of calling partner* (*real or broadcasted*) *affect the call timing of individual pups?*’ (Q2) and ‘*when the focal pup is vocalizing, does the presence of a silent partner* (versus *no partner*) *affect call timing?*’ (Q3). We answer these questions using circular statistics (Q2 and Q3) and Granger causality tests (Q2). Lastly, ADAM is used to investigate the fourth question about the cognitive processes involved in temporal adjustment: *‘which timing mechanisms are used by pups during vocal interactions?’* (Q4).

**Table 1.** Summary table showing in order: research questions, analyses, contributing pups, predictions, whether the data supports each prediction, statistical test(s) used and result(s) obtained. (The column ‘supported by data?’ has three possible answers: results support the prediction (Y), results only partially support the prediction (partial) and results do not support the prediction (N ). Owing to sample size and/or analytical requirements, not all pup data could be used in each analysis. The acoustic variables of interest for the different analytical approaches were response phases (circular statistics), inter-onset intervals (IOI) ratios (categorical rhythms), IOIs (Granger causality, ADAM) and asynchronies (ADAM). SIR, small integer ratio; KS, Kolmogorov–Smirnov.)

Research question	Analytical approaches	Pups	Prediction	Supported by data?	Statistical test(s) and result(s)
***temporal domain, Q1:*** *does the presence of a calling partner affect the call timing of individual pups?*	circular statistics	A–I	pups will not vocalize at random points in time	Y	Rayleigh test: unimodal distribution of response phases
			pups will call in asynchrony to avoid overlap during vocal interactions	Y	V-test: pup calls start at one-quarter of the partner's period
			response phases will be affected by the presence of a vocalizing partner	Y	Watson's U^2^ test: response phase distributions differ between non-interactive and interactive contexts
	categorical rhythms	A, B, C, E, H, I	empirical and chance ratio distributions will only significantly differ when pups are vocally interacting	partial	one-sample KS tests: simulated and empirical ratio distributions are rarely significantly different (exceptions: pup I alone, pup I one-way, pup B two-way)
		A, B, I	vocally interacting pups will have a significant peak at the 4 : 1 SIR	N	paired Wilcoxon signed-rank test: no significant peaks at any of the tested SIRs
			ratio distributions of individual pups will differ across behavioural contexts	Y	two-sample KS tests: ratio distributions significantly differed for the same individuals in different behavioural contexts
***social domain, Q2:*** *does the type of calling partner* (*real or broadcasted*) *affect the call timing of individual pups?*	circular statistics	A–I	pups interacting with a real partner will show more adaptive call timing than the pup interacting with a broadcasted partner	N	Watson's U^2^ test: no difference in response phases between one-way and two-way interactions
	Granger causality	A, B, C, D, E, F, I	the time series of a pup will be better predicted considering the time series of a vocalizing partner rather than the time series of a broadcasted signal	Y	bidirectional and unidirectional causality: interaction with a real partner impacted the pup's vocal behaviour more than the playback. Mutual temporal adaptation among pairs of vocally interacting pups
***social domain, Q3:*** *when the focal pup is vocalizing, does the presence of a silent partner* (versus *no partner*) *affect call timing?*	circular statistics	A–I	the calling pattern of pups will show similar rhythmic structure in both the alone and silent partner conditions, as no vocal interaction is taking place in both cases	Y	Watson's U^2^ test: no difference between response phase distributions of pup calling alone and pups calling with a silent partner
***cognitive domain, Q4:*** *which timing mechanisms are used by pups during vocal interactions?*	ADAM	A, B, I	in the one-way interaction, pups may show sensitivity to (non-) interactivity which would be reflected by parameter changes over repeated sessions	partial	one-way interaction: temporal anticipation, and to a lesser extent also adaptation, decreased across the playback sessions, and was absent in the final session
			the two-way interaction may be mediated by basic temporal adaptation and possibly higher level anticipatory timing		two-way interaction: clear evidence for temporal adaptation, with differing parameter estimates for each seal pup suggesting the emergence of different interactive roles

### (e) Subjects, housing conditions and behavioural contexts

We recorded nine wild-born pups (A–I) calling in different behavioural contexts while housed at Sealcentre Pieterburen (the Netherlands) (electronic supplementary material, Methods S1 and S2). During the recordings, each pup was housed in an enclosure with a swimming pool and a resting platform (electronic supplementary material, figure S1). One pup was housed alone (I) while the others were housed in pairs (A/B, C/D, E/F, G/H). Note that the enclosures were physically but not acoustically isolated from each other, meaning that pups could hear other pups in neighbouring enclosures.

**Figure 1.**
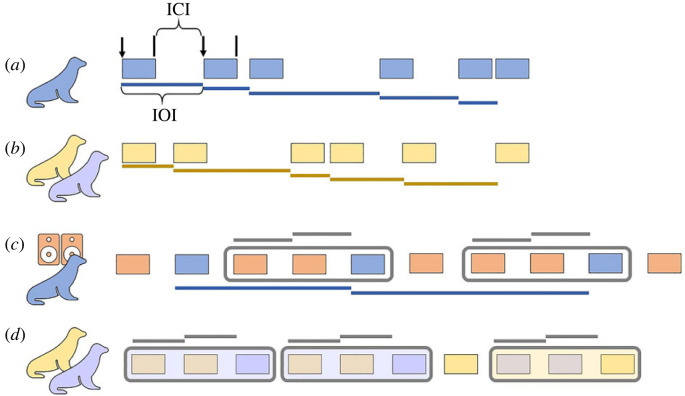
Schematic of experimental conditions and key measurements. Each of the four rows represents one bout and indicates a different behavioural context: (*a*) alone, (*b*) silent partner, (*c*) one-way interaction, and (*d*) two-way interaction. Boxes are coloured to represent the call source and grey rectangles denote vocal interactions. For bouts (*c*) and (*d*), calls are isochronously spaced for ease of visualization, but empirical patterns were not necessarily isochronous. Horizontal bars indicate how inter-onset intervals (IOIs) were calculated for different analyses. The call onsets (black arrows) and offsets (black lines) are shown for the first two calls in (*a*). ICI, inter-call interval.

We analysed focal pup vocalizations during four different behavioural contexts (figure 1; electronic supplementary material, table S1): (i) when the focal pup was alone (pup I); (ii) when the focal pup heard a playback of conspecific calls (pup I); (iii) when the focal pup's partner was silent (pups A–H); and (iv) when the focal pup's partner was also vocalizing (pups A–H). Hereafter, we refer to these conditions as: (i) alone, (ii) one-way interaction with a broadcasted partner, (iii) silent partner, and (iv) two-way interaction with a real partner. Notice that only some pups entered each condition and vice versa (electronic supplementary material, table S1).

Our sample size was affected by the unpredictable arrival of animals at the Sealcentre, which varies seasonally. Only medically stable and healthy pups were included in this study. The number of daily recording sessions per pup pair varied based on veterinary staff recommendations at the rehabilitation centre (electronic supplementary material, table S1); we did not record pups with signs of disease. Data from pup I (i.e. the alone and one-way contexts both with and without playback stimuli) have been re-analysed from previous studies ([20,27], respectively) and combined with unpublished data from pups A–H (the silent partner and two-way contexts) (electronic supplementary material, table S1). The contribution of each pup to each analysis is shown in the electronic supplementary material, table S2.

### (f) Extraction of temporal variables, definition of call bout and vocal interactions

We first extracted the onsets and offsets of each pup call recorded in each behavioural context (figure 1; electronic supplementary material, Method S3). From these values, we calculated rhythmic metrics such as call duration, inter-onset intervals (IOIs), ratios of adjacent IOIs and inter-call intervals (ICIs; i.e. silent gaps). Each IOI was obtained by subtracting the *onset* of call *n* from the *onset* of call *n* + 1, while the ICI was calculated by subtracting the *offset* of call *n* from the *onset* of call *n* + 1 (i.e. IOI minus duration of call *n*). Calls were organized into bouts, defined here as a series of at least three subsequent calls that were separated from adjacent bouts by a period greater than 1.5 times the median ICI of the recorded individual(s) calls (figure 1). The response phase was computed as the ratio of the ‘response IOI’ (i.e. time interval between the call onset of the partner and the call onset of the pup's response) and the previous IOI, multiplied by 360, resulting in a unit vector with an angle on a circle. A vocal interaction was defined as a group of three calls within the same bout, which includes two calls from the broadcasted/real partner followed by the response of the focal individual (figure 1). Following previous methodology [27,37], we calculated the IOI ratio, *r_k_*, for each pair of adjacent IOIs, *t_k_* and *t_k_*_+1_, in a bout as:

$$r_{k}=\frac{t_{k}}{t_{k}+t_{k+1}}.$$

### (g) Descriptions of analytical approaches

**Circular statistics,** wherein periodic measures are converted to angles on a circle and compared to distributions of interest [39], were used to investigate rhythmic periodicities in pup call response phases (electronic supplementary material, Method S4). Following [20], we considered the values of the response phases as circular data falling between 0° and 360°. We obtained the circular mean (*μ*) (i.e. the average direction of the response phases calculated from the pup calls; electronic supplementary material, table S3). Then, we ran Rayleigh *z*-tests to investigate whether the distribution of response phases was uniform (e.g. arousal hypothesis) or showed a unimodal peak (electronic supplementary material, table S4) [20]. Subsequently, we tested for uniformity against a specified mean direction for the unimodal peak using a V-test [39–42].

Our data met the assumptions for circular statistics. We tested if the response phases in all four behavioural contexts followed a von Mises distribution using one-sample Watson tests (electronic supplementary material, table S5). With deviations from uniformity (null hypothesis von Mises distribution rejected), we used Kuiper's test, Watson's test and Rao's spacing test, to confirm the *p*-value obtained from the Rayleigh test (electronic supplementary material, table S6) as suggested by Landler *et al*. [39]. More details are shown in the electronic supplementary material, Method S4.

We then tested whether response phase distributions varied depending on the presence of the calling partner. We expected that vocally interacting pups would adjust their responses to broadcasted (one-way interaction) or real (two-way interaction) conspecific calls to avoid overlap and, hence, their response phases would show a unimodal distribution. Following previous work [20] and applying the V-test, we tested the null hypothesis of call response phase uniformity against two alternative unimodal departures: 0° (i.e. synchrony) and 90° (i.e. asynchrony). Using Watson's two-sample U^2^ test [43], we also compared the call phase distributions of: (i) a pup calling alone versus when responding to a broadcasted partner (pup I), and (ii) a pup calling in the presence of a silent partner versus when their partner was also calling (pups A–H). For interacting (one-way or two-way) pups, we applied Watson's two-sample U^2^ test to assess whether the type of partner (i.e. real or broadcasted) differentially affected the pups' response timing. We predicted that pups interacting with a real partner would show more adaptive call timing, thanks to potential communicative cues from other modalities. Lastly, we compared the distributions of call phases of the single pup vocalizing alone to those of the paired pups when their partner was silent to test whether the simple presence of a silent partner affects individual call timing. In both behavioural contexts, we predicted that calling patterns for pups without a responsive partner would show a different rhythmic structure to those observed in interaction.

**Categorical rhythm** analysis tests whether the temporal intervals between signal onsets, as inferred from IOI ratios, are distributed categorically rather than uniformly. We predicted that empirical and simulated null ratio distributions (i.e. the expected distribution if no rhythmic categories exist) will not differ when a pup is alone or with a silent partner but will differ when a pup is vocally interacting. For vocally interacting pups, we predicted a significant peak in empirical ratio distributions at the 4 : 1 small integer ratio (SIR) based on the lone seal in [20], which called at approximately one-quarter of the playback's period. All categorical rhythm analyses were done within bouts following previous methodology (electronic supplementary material, Method S5) [22,23], with IOIs calculated in various ways depending on the behavioural context (electronic supplementary material, table S8; figure 1). We used one-sample Kolmogorov–Smirnov (KS) tests to determine whether empirical IOI ratio distributions significantly differed from simulated null IOI ratio distributions. Our data met the one-sample KS test assumptions, namely that the sample is random and the theoretical distribution is continuous and fully defined. When the empirical and simulated distributions were significantly different, we also looked for evidence of SIR categorical rhythms—specifically at the 1 : 4, 1 : 3, 1 : 2, 1 : 1, 2 : 1, 3 : 1, and 4 : 1 ratios—which have been found in other species' vocalizations [22,23]. In these analyses, the empirical ratio distributions were divided into ‘on-integer’ and ‘off-integer’ ratio bins (electronic supplementary material, table S7). On- and off-integer bin counts for each SIR were normalized by bin size and compared using a paired Wilcoxon signed-rank test (a non-parametric test that allows for non-normality in the population data and assumes paired differences are continuous, symmetrically distributed and mutually independent). When sample sizes allowed, we used two-sample KS tests (having met the assumption of mutual independence of measurements within samples) to determine whether the ratio distributions of individual pups differed across behavioural contexts.

The **Granger causality** test investigates whether the values of a time series A are better predicted when considering the values from a second time series B, as opposed to only using values from time series A [44]. Here, we assessed whether the call timing of a pup partaking in a one-way or two-way vocal interaction can be predicted using the call timing of its partner. More specifically, to investigate whether the call timing of a pup differed in relation to the type of partner, we tested whether there is a difference in predicting the time series of the pup interacting with a broadcasted partner versus time series of the pups interacting with a real partner. Previous work showed that individuals respond to conspecific calls with a non-random pattern [17–19,45]. We therefore expected that the time series of a pup can be better predicted considering the time series of a vocalizing partner rather than those of a broadcasted signal. In both the one-way and two-way interactive scenarios, we considered Granger causality at two levels: (i) the entire recording, regardless of the length of the pauses between consecutive calls, and (ii) different bouts within each recorded session. We restricted the analysis on the different bouts to call sequences that were long enough to generate accurate estimates (i.e. a minimum of five paired calls [46]). The bouts included in this analysis range from 5 to 20 calls. We conducted the Granger causality test using call onsets and different *lag* measures, from one to five (electronic supplementary material, Method S6), testing whether the previous one to five onsets in the first time series can be used to better predict the second time series (electronic supplementary material, figures S5 and S6, and table S11). For the one-way interaction, we performed a one-way analysis, considering whether the pup's timing could be predicted using the playback timing. For the two-way interaction, we performed a two-way analysis to assess whether the two interacting pups influenced the timing of each other's calls.

We used **ADAM** to test for evidence of reactive error correction and predictive processes in the one-way and two-way interactive scenarios. ADAM consists of three computational modules that interact via internal models of ‘self’ and ‘other’ that support one's own action planning and external event prediction, respectively (electronic supplementary material, figure S2). The *adaptation module* compensates for synchronization errors by implementing error correction processes that alter the phase and/or period of an internal timekeeper controlling for action (here, call) timing. These error correction processes determine the provisional timing of the next planned action by providing input to an internal model of the ‘self’. The *anticipation module* computes the expected timing of upcoming events based on the weighted sum of two processes: the linear extrapolation of previous IOIs in the sequence and the copying (or ‘tracking’) of the previous IOI, with the output informing temporal predictions generated by the ‘other’ internal model. Finally, a *joint module* integrates and compares the output of the adaptation and anticipation modules and compensates for discrepancies by implementing an anticipatory error correction process before the next motor command is issued. The joint module thus reduces potential temporal mismatches between action plans in ‘self’ internal models and temporal predictions in ‘other’ internal models, thereby regulating the balance between the integration (merging) and segregation (distinction) of information about ‘self’ and ‘other’ [47,48]. Each process instantiated in ADAM is controlled by an independent parameter, and the value of these parameters can be estimated for a particular individual by fitting the model to behavioural time-series data [25,26,49,50]. Parameter estimates were obtained for both the adaptation-only version of ADAM—which includes phase correction and period correction—and the full (joint) version—including period correction, temporal prediction-tracking and anticipatory error correction. Both versions of ADAM were applied to each interactive context because it is not possible to know *a priori* whether the pups' call sequences (real or broadcasted) have a steady base tempo (for which adaptation is sufficient) or a systematically changing tempo (which benefits from both anticipation and adaptation) (electronic supplementary material, Method S7).

## 3. Results

### (a) Does the presence of a calling partner affect the call timing of individual pups? (Q1)

**Figure 2.**
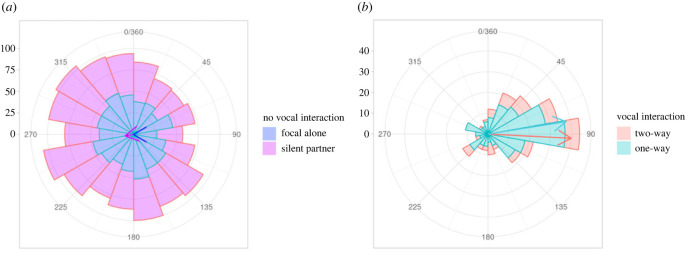
Circular histogram plots (bin width = 20°) showing response phases in (*a*) both types of behavioural contexts with no vocal interaction (alone/silent partner), and (*b*) in both types of vocal interaction contexts (one-way/two-way). Angles are measured in degrees starting from 0° and going clockwise to 360°. The arrows indicate the circular mean (*µ*) and colours correspond to the different behavioural contexts. The length of the arrow corresponds to the value of the mean resultant length (*ρ*).

All pups’ data entered the circular statistics analysis. Running the Rayleigh test, we found that the response phase distribution was uniform for pup I which was recorded alone (*z* = 0.04, *p* = 0.254; figure 2*a*), whereas it was non-uniform for pups A–H which were recorded with a silent partner (*z* = 0.11, *p* < 0.001; figure 2*a*; table 1). This non-uniformity may have been driven by the individual contributions of pups B and C, which had non-uniformly distributed response phases (electronic supplementary material, table S4), whereas the other six pups had a uniform distribution. The Rayleigh tests run anew in the interactive contexts, showed that the response phase distributions of pup calls were unimodal in both the one-way (pup I: *z* = 0.39, *p* < 0.001) and two-way (pups A–H: *z* = 0.41, *p* < 0.001) interactions (figure 2*b*; table 1). Applying the V-test in both contexts, the direction of the response phases did not statistically match 0° (one-way: *z* = −0.02, *p* = 0.587; two-way: *z* = 0.06, *p* = 0.110), suggesting that pups did not synchronize with their partner (real or broadcasted). However, the response phase direction did match 90° (one-way: *z* = 0.38, two-way: *z* = 0.41, *p* < 0.001), supporting the previously reported evidence of asynchronous calling behaviour [20]. A Watson's two-sample U^2^ test confirmed that the response phase distributions significantly differed between the alone versus one-way interaction context for pup I (U^2^ = 1.76, *p* < 0.001), and between the silent partner versus two-way interaction context for pups A–H (U^2^ = 0.78, *p* < 0.001; electronic supplementary material, figure S3; table 1). Finally, the circular standard deviation values were higher for the alone and silent partner contexts compared to both interactive vocal contexts, indicating a larger dispersion of the response phases for the former conditions. This outcome is also confirmed by the values for the mean resultant length.

**Figure 3.**
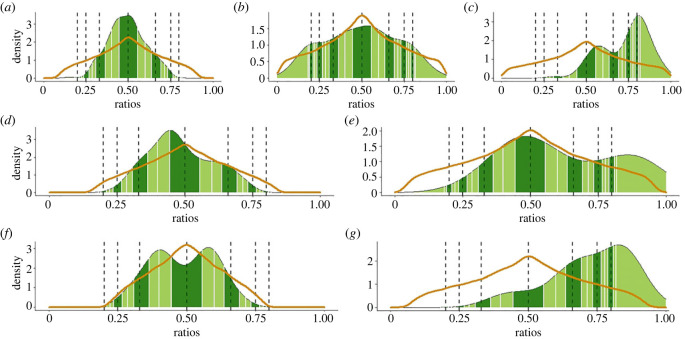
IOI ratio density plots for pups in different behavioural contexts. Pup I: (*a*) alone, (*b*) one-way interaction (disregarding playback), (*c*) one-way interaction (responding to playback). Pup A: (*d*) silent partner, (*e*) two-way interaction (responding to partner). Pup B: (*f*) silent partner and (*g*) two-way interaction (responding to partner). For each plot, the dashed vertical lines indicate, from left to right, 1 : 4, 1 : 3, 1 : 2, 1 : 1 (i.e. isochrony), 2 : 1, 3 : 1 and 4 : 1 SIRs. On-integer ratio ranges are in dark green and off-integer ratio ranges are in light green, with white lines and black dashed lines denoting bin boundaries. The orange curves indicate the ratio distribution expected under a uniform distribution if no rhythm categories exist. The empirical ratio distribution significantly differed from the simulated ratio distribution for (*a*), (*c*) and (*g*) only. Note that the scale of the *y*-axes differ.

In the categorical rhythm analyses, the empirical ratio distribution was significantly different from chance when pup I was recorded alone (figure 3*a*) and during the one-way interaction (figure 3*c*). In both contexts, there was no evidence of significant peaks at any of the tested ratios (table 1). When the playback calls were disregarded from IOI calculations (figure 3*b*), there was no significant difference in empirical and simulated ratio distributions for pup I. However, pairwise KS tests showed that the ratio distributions significantly differed when comparing each of the three behavioural contexts (alone versus one-way interaction disregarding playback versus one-way interaction when pup I responds) to each other (electronic supplementary material, table S10; table 1). For the five pups that were well-sampled in the silent partner context (pups A, B, C, E and H; electronic supplementary material, table S8), the empirical ratio distributions did not significantly differ from chance (figure 3; electronic supplementary material, table S9 and figure S4, Method S5). Finally, when considering two-way interactions, only pups A and B were well-sampled enough (i.e. had at least 10 ratios for both the silent partner and two-way interaction contexts) to compare, but the empirical and simulated ratio distributions were significantly different only when pup B was the responder (figure 3; electronic supplementary material, table S8 and table S9). Once again, there were no significant peaks at any of the tested ratios for pup B. For both pups A and B, the empirical ratio distributions significantly differed when comparing different behavioural contexts (electronic supplementary material, table S10). Collectively, there was thus little evidence of SIR rhythmic categories in pup calls, but IOI ratios did significantly differ when looking at the same individuals in different behavioural contexts (table 1).

Thus, the results from both analyses suggest that the *presence* of a calling partner does affect the call timing of the focal pup, in terms of both call response phases and IOI ratios.

### (b) Does the type of calling partner (real or broadcasted) affect the call timing of individual pups? (Q2)

To address this research question, we once again used circular statistics and the response phase distributions of all nine pups. Specifically, we compared calls from pup I during the playback (one-way interaction) with calls of pups A–H when their partner was also calling (two-way interaction). A Watson's two-sample U^2^ test statistically confirmed that the response phase distributions did not differ between the one-way and two-way vocal interactions (U^2^ = 0.07, *p* > 0.10; table 1).

Interestingly, however, the Granger causality results from seven pups (A–F, I) showed that call timing behaviour differed depending on the type of partner (table 1). For the one-way interaction, five different playback sessions featuring pup I were considered (ranging from 34 to 121 paired calls) and the timing of the pup's calls were never significantly predicted by the timing of the playback (electronic supplementary material, table S11 and figure S5). For the two-way interaction, five different recording sessions were considered (ranging from 8 to 71 paired onsets). We found that in two sessions, the timing of the first pup did not significantly predict the timing of the second, in either direction (pair A > B and B > A; pair E > F and F > E; electronic supplementary material, table S11 and figure S6B and S6D). However, in two other sessions, the timing of a pup was significantly predicted by the calling partner in both directions, across different lag values (A > B: lag-1, lag-3, lag-4, lag-5; B > A: lag-3, lag-4, lag-5; C > D and D > C: lag-2 and lag-3; electronic supplementary material, table S11 and figure S6B and S6C). In the last session, the timing of pup A could be predicted by that of pup B, while we found no indication of temporal adjustment for pup B. At the bout level, we restricted our analyses to four series of paired onsets featuring pups A and B (as they were the only pup pair that met the sample size requirements). In this last scenario, only the timing of pup B was significantly influenced by pup A (electronic supplementary material, figure S6A).

While the circular statistics results thus suggest that the type of calling partner (real versus broadcasted) does not affect focal pup call timing, the Granger causality results suggest that in certain two-way (but not one-way) interactions, focal pup call timing can be predicted by the partner's call timing.

### (c) When the focal pup is vocalizing, does the presence of a silent partner (versus no partner) affect call timing? (Q3)

Using circular statistics, we compared the calls of pup I recorded alone with those of the eight other pups (A–H) recorded with a silent partner present. Watson's two-sample U^2^ test results show that the response phase distributions did not statistically differ between the two contexts (U^2^ = 0.10, *p* > 0.05). In other words, having a silent partner was essentially the same as having no partner—in terms of the effect on focal pup response phase distributions—for the pups in our study. This is intuitive, given that in both behavioural contexts, there is no acoustic stimuli to ‘respond’ to.

### (d) Which timing mechanisms are used by pups during vocal interactions? (Q4)

**Figure 4.**
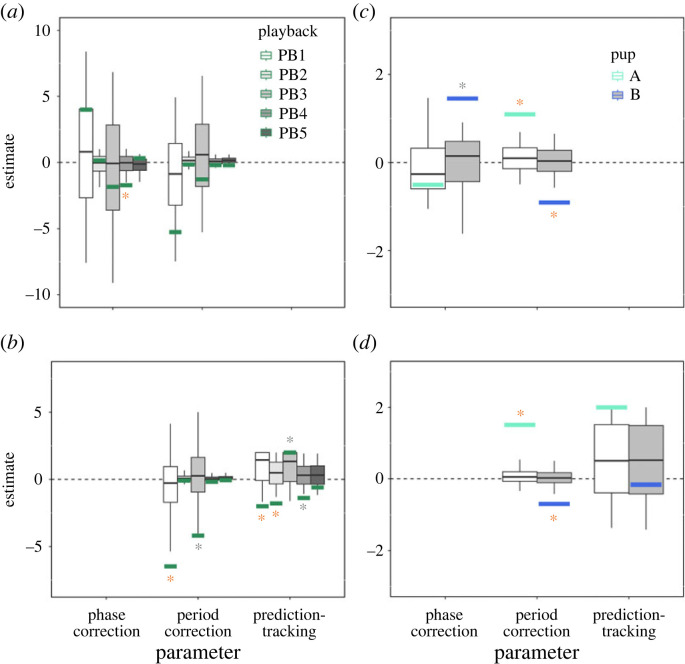
ADAM parameter estimates for seal pups A, B and I. (*a*,*b*) Parameter estimates for pup I in five playback sessions (one-way interaction); (*c*,*d*) estimates for pups A and B (two-way interaction). (*a*) Phase correction and period estimates obtained with the adaptation-only version of ADAM; (*b,d*) period correction and prediction-tracking estimates for the full ‘joint’ model (anticipatory error correction estimates are not shown). Parameter estimates are shown separately for pup A relative to pup B (aquamarine bars) and pup B relative to pup A (blue bars) in the recording session, and for pup I (green bars) relative to playback calls in separate playback sessions (PB1–PB5). Corresponding parameter estimates for randomly permuted data from each session are represented by box plots, with the central horizontal line indicating the median, the bottom and top edges of the box indicating the 25th and 75th percentiles, and the whiskers indicating the 5th and 95th percentiles. Real estimates with asterisks are significantly different from permuted data estimates at the two-tailed (orange) or one-tailed (grey) level.

ADAM parameter estimation was conducted on call data from all five playback sessions featuring pup I (one-way, figure 4*a*,*b*), but was restricted to pups A and B for the two-way interactions owing to sample size limitations (figure 4*c*,*d*). Interactive vocal bouts were concatenated to obtain a time-series length which would provide reliable ADAM parameter estimates. A simulation test then ensured that the estimates were not compromised by differing sequence lengths or by the concatenation procedure (electronic supplementary material, Method S7). The reliability of observed parameter estimates was tested by comparison against corresponding values for randomly permuted data (electronic supplementary material, Method S7). The quality of the fits to the data did not differ significantly between versions of ADAM (electronic supplementary material, Method S7). Results for the one-way interaction featuring pup I were remarkable with regard to typical human data (e.g. [25,26]) as most significant parameters were negative in sign (figure 4*a*,*b*; electronic supplementary material, tables S13 and S14). Negative phase and period correction estimates indicate that calling earlier will lead to a shortening of the next IOI, while calling later will lead to a lengthening of the next IOI. Negative prediction-tracking estimates mean that when the playback's IOIs increase (i.e. deceleration), the pup's IOIs will decrease (i.e. acceleration), and vice versa. This systematically enhances the timing distinction between calls, possibly testing the responsiveness of the partner (i.e. playback) by introducing timing asynchronies and gauging their effects. It is worth noting that evidence of such behaviour generally decreased across the five playback sessions, with no significant parameter estimates emerging in the final session.

For the two-way interaction (figure 4*c*,*d*; electronic supplementary material, tables S13 and S14), the parameter estimation procedure was run twice, each time with one of the two pups serving as the focal individual and the other as the external reference. With this procedure, similar parameter estimates for each pup would indicate a symmetrical pattern of influence, whereas different estimates would indicate asymmetrical influence. Results for this type of vocal interaction showed evidence for temporal adaptation. In particular, parameter estimates were consistent with pup A engaging in period correction while pup B engaged in both phase and period correction. Period correction estimates were positive in sign for pup A and negative for pup B. Pup A's positive period correction estimates suggest that calling earlier will lead to a lengthening of the next IOI, whereas calling later will lead to a shortening of the next interval. Pup B exhibited the opposite effect but to a lesser degree. Pup A thus implemented a timing mechanism that increased the similarity in their calling rates, while pup B implemented a timing mechanism that increased the distinction in calling rates. It should be noted, however, that the observed parameter estimates do not necessarily reflect individual call styles per se, but rather the roles that dynamically emerge within the context of this specific pairing of pups.

## 4. Discussion

This study sought to provide a methodological proof of concept for quantifying vocal rhythmicity in non-human animal interactive communication. Particularly, we showed how complementary methodologies can be used to test whether the (Q1) presence and (Q2) type of a vocalizing partner, or the (Q3) presence of a silent partner affect patterns in animal communication (table 1). We also investigated which (Q4) underlying cognitive mechanisms potentially play a role in vocal interactions (table 1). The different analytical techniques proved fruitful; combining and contrasting their results could highlight nuances in rhythmic behaviour. Nonetheless, our sample size is undeniably small, and our opportunistic design (e.g. mixing within- and between-individual comparisons) cannot provide strong inference. We believe that our experimental set-up, combined with the approaches we present, can be adapted and expanded in future work to better understand the temporal, social and cognitive processes underlying interactive communication in animals.

### (a ) Q1. The presence of a vocalizing partner influences rhythm in vocal interactions

The prediction of overlap avoidance was supported by circular statistics, which showed that the distributions of response phase angles were unimodal. When vocally interacting, pups responded at approximately one-quarter of the playback/real partner calling period (90°) and showed phase angles significantly different from 0°, supporting previous results from one individual (pup I) [20]. Pups responding to conspecifics seem to time their calls to avoid overlap, consistently de-synchronizing their call onsets. When pups were recorded alone or with a silent partner, the distribution of phase angles was uniform, with no significant rhythmic pattern. Our findings confirm and extend previous outcomes [20]; in our limited sample, pups not only react to playbacks with asynchrony, but also respond to conspecific partner calls in an asynchronous manner.

Regarding categorical rhythms, the empirical ratio distribution did not significantly differ from the distribution expected by chance if IOIs were uniformly distributed for most pup/behavioural context combinations (7 out of 10). For the remaining three combinations (pup I alone; pup I, one-way interaction; pup B, two-way interaction), there was no significant evidence that call timing exhibited categorical rhythms at the seven tested SIRs. This includes the 4 : 1 ratio, which we hypothesized would frequently occur when pups were vocally interacting with playback stimuli or partners based on previous work [20]. Importantly, however, the empirical ratio distributions significantly differed across behavioural context conditions for the three pups (A, B, I) for whom such comparisons were possible. When alone or with a silent partner, pup calls generally showed unimodal ratio distributions centred around isochrony, whereas calls emitted by vocally interacting pups showed a clear right-shifted peak, or a second peak to the right of isochrony, indicating that the pup's IOIs during vocal interactions were generally shorter than the playback's/partner's IOIs. This context-dependent asymmetry bolsters results related to Q4, namely that the presence of vocalizing partner can significantly impact pup vocal behaviour, with interacting individuals trying to prevent call overlap. Roeske *et al*. [22] hypothesized that categorical rhythms may be prevalent in calls meant to attract and hold conspecific attention, such as the calls pups use to attract the attention of their mothers. We did not find evidence of rhythm categories at seven tested ratios; this negative result has methodological value, highlighting that not all species which produce attention-seeking vocalizations show integer ratio categories.

A vocal interaction with non-adaptive playback stimuli (one-way) may represent a limitation to studying spontaneous vocal production. The extent of this limitation can be gauged by comparing results to data from vocal interactions with a real partner (two-way), and with the use of complementary analytical methodologies, such as categorical rhythm analysis and circular statistics.

### (b) Q2. Type of calling partner (real or broadcasted) partly affects rhythm in interaction

Findings from Q2 show contrasting results. Call phases were statistically the same no matter if pups could interactively adjust their call timing to each other (two-way) or not (one-way). Partly in contrast with this, the Granger causality analysis showed how vocally interacting with a real individual impacted the pup's vocal behaviour more than interacting with a broadcasted partner. Indeed, we found evidence of mutual temporal adaptation among pairs of vocally interacting pups and, consistent with a recent hypothesis [6], conspecific interactions can be facilitated by the reciprocal adjustment of timing behaviour. By contrast, we found little evidence that a pup adjusts the time series of its calls to a playback series. Indeed, in most cases, the pup's call timing could not be predicted by the playback's call timing.

Together, findings from circular statistics (Q1) and Granger causality (Q2) point towards a directionality-overlap avoidance relationship, which has also been suggested for other non-human species (e.g. primates [17,51]; birds [52]; amphibians [53] and seals [54]). Interestingly, when infant marmosets interact with their parents, the probability that their vocalizations will overlap with those of adults decreases over time [21], suggesting that turn-taking in some mammals is a learned vocal behaviour scaffolded by active parental feedback [38].

### (c) Q3. A silent partner does not trigger call rhythmicity

Circular statistics indicated that pups did not show any periodic pattern when calling alone or with a silent partner present (Q1). This is consistent with the fact that wild pups produce calls to attract a silent mother [35]: if no other calling conspecifics are present, there is no need to adjust one's call timing and vocalizations are produced with a random onset. Moreover, given that the response phase distributions did not differ between pups recorded with a silent partner or alone, it suggests that the presence of a silent partner did not trigger variable calling behaviour in our study population.

### (d) Q4. Purported timing mechanisms for vocal interactions

The ADAM analysis suggests that seal pups may perceive temporal patterns [55], which arise between their calls and those of others, and react to them by adopting different mechanisms for temporal adaptation. The negative parameter estimates that we observed in the one-way interaction sessions (consistent with enhancing the distinction between calls) could reflect attempts to lead the temporal interaction or even to test the responsiveness of the (broadcasted) partner by introducing timing perturbations and gauging their effects. The decrease of temporal adaptation and/or anticipation observed across playback sessions is consistent with a gradual process of habituation, with the pup possibly learning that the playback is non-interactive. Harbour seals are capable of acoustic recognition based on habituation paradigms; they can discriminate among different stimuli and selectively habituate to them [56]. The lack of temporal adaptation to the playback stimulus we observed in a seal pup may entail similar habituation processes. From a comparative perspective, these findings also suggest a sensitivity to social contingency that may be analogous to capacities in human infants, who become disinterested and display fewer signs of positive affect during vocal interactions with non-responsive or delayed video recordings of their mothers [57–61]. Future studies could address the role of temporal contingency by using interactive playback sequences [62–64].

Ours constitutes, to our knowledge, the first attempt to apply ADAM to non-human animals. On the technical side, this necessitated the validation of an approach where brief interactive vocal bouts were concatenated to produce longer time series and thereby reduce the risk of model overfitting (electronic supplementary material, Discussion S1). Demonstrating the use of this procedure opens the door to applying the model in a wider range of behavioural contexts. Nevertheless, caveats are necessary when interpreting the seal data in light of previous work with ADAM in humans, where individuals intentionally produce movements, whose sensory effects occur simultaneously with rhythmically regular sounds (e.g. [25,26]). Assumptions about intentionality and simultaneity may not apply to seal pup vocal interactions or rhythmic interactions in other animals [6,9]. Points of convergence in the main outcomes of the complementary analysis techniques suggest that ADAM, like categorical rhythm analysis, circular statistics and Granger causality, may also be robust and informative under such conditions.

## 5. Conclusion

Crucially, our work highlights the efficacy of combining multiple methods to study rhythmic vocal behaviour. Our approaches vary in the degree to which they capture global temporal characteristics across events versus local temporal dependencies between events. Global measures (e.g. from circular statistics or categorical rhythm analysis) reveal predominant rhythmic features of a vocal interaction while local time-series measures (e.g. from Granger causality or ADAM) provide information about how these features might arise. Through this approach we could tease apart rhythm nuances in our dataset, further develop harbour seals as a model species, and demonstrate how certain analyses often restricted to humans, such as categorical rhythms (but see [22,23]) and ADAM, can be applied to non-human animals. The categorical rhythm and circular statistical analyses tackled similar questions from different angles, namely whether the distributions of IOI ratios (the former) or call response phases (the latter) significantly differed across behavioural contexts. The categorical rhythm analyses also sought to determine whether the rhythmicity of pup calling behaviour conforms to SIRs. Our negative result is, to our knowledge, the first published case of a species for which categorical rhythms are clearly absent from vocalizations, which adds to understanding of how, why and when such rhythms evolve in communication systems. Time-series analyses such as Granger causality allowed testing for timing adjustment. In the case of ADAM, mechanisms of temporal adaptation and anticipation that have previously been used to describe rhythmic behaviour in humans [65] were used to describe rhythmic behaviour in seals. Interestingly, the ADAM model provides an empirical warning about potential seal pup habituation effects when vocally interacting with a recorded partner.

Though our sample size is small, studies on single individuals are not unusual in comparative research [19,66]. Nevertheless, it is possible that the lack of adjustment to a playback, the adaptation to a real individual, or both, reflect a peculiar vocal behaviour of the individuals we tested and cannot be generalized to the species as a whole. The pups in this study were in a temporarily captive setting, albeit in acoustic proximity to other individuals, similar to conditions they would experience in nature. Unfortunately, vocal development in harbour seal pups has not been studied in wild colonies, hence we do not know whether captivity affects their vocal development. We do know, however, that pups vocally interact with neighbouring pups in the colony and not with their silent mothers; hence the turn-taking behaviour observed in our captive conditions might extend to the same behaviour in wild conspecifics.

Motivation for an individual to respond and engage in an interaction, with the closest partner in the colony, may depend on the degree of participation signalled by the partner. This, in turn, may be triggered by individual-specific behaviours or by cues from other modalities. Multi-modal communication should be the target of future studies since we cannot assume that such interactions are limited to acoustic cues. Despite these limitations, our study shows that adopting multiple complementary approaches can be a fruitful way to study rhythmic interactivity in non-human animal communication.

## Data Availability

The datasets and codes for statistical analysis used in this article have been uploaded as part of the electronic supplementary material [67]. Please follow this link to find the related files: https://osf.io/8m4yv/.

## References

[1] Dusenbery DB. 1992 Sensory ecology: how organisms acquire and respond to information. New York, NY: Freeman.

[2] Stegmann UE. 2013 Animal communication theory: information and influence. Cambridge, UK: Cambridge University Press.

[3] Anichini M, de Heer Kloots M, Ravignani A. 2020 Interactive rhythms in the wild, in the brain, and *in silico*. Can. J. Exp. Psychol. **74**, 170-175. (10.1037/cep0000224) 33090846

[4] Ravignani A, Bowling DL, Fitch WT. 2014 Chorusing, synchrony, and the evolutionary functions of rhythm. Front. Psychol. **5**, 1118. (10.3389/fpsyg.2014.01118) 25346705 PMC4193405

[5] Greenfield MD, Aihara I, Amichay G, Anichini M, Nityananda V. 2021 Rhythm interaction in animal groups: selective attention in communication networks. Phil. Trans. R. Soc. B **376**, 20200338. (10.1098/rstb.2020.0338) 34420386 PMC8387861

[6] de Reus K, Soma M, Anichini M, Gamba M, de Heer Kloots M, Lense M, Bruno JH, Trainor L, Ravignani A. 2021 Rhythm in dyadic interactions. Phil. Trans. R. Soc. B **376**, 20200337. (10.1098/rstb.2020.0337) 34420383 PMC8380972

[7] Levinson SC. 2006 Cognition at the heart of human interaction. Discour. Studies **8**, 85-93.

[8] Levinson SC. 2006 On the human ‘interaction engine’. In Roots of human sociality: culture, cognition and interaction (eds NJ Enfield, SC Levinson), pp. 39–69. Oxford, UK: Berg.

[9] Ravignani A, Verga L, Greenfield MD. 2019 Interactive rhythms across species: the evolutionary biology of animal chorusing and turn-taking. Ann. N Y Acad. Sci. **1453**, 12-21. (10.1111/nyas.14230) 31515817 PMC6790674

[10] Levinson SC. 2016 Turn-taking in human communication–origins and implications for language processing. Trends Cogn. Sci. **20**, 6-14. (10.1016/j.tics.2015.10.010) 26651245

[11] Pika S, Wilkinson R, Kendrick KH, Vernes SC. 2018 Taking turns: bridging the gap between human and animal communication. Proc. R. Soc. B **285**, 20180598. (10.1098/rspb.2018.0598) PMC6015850 29875303

[12] Demartsev V, Strandburg-Peshkin A, Ruffner M, Manser M. 2018 Vocal turn-taking in meerkat group calling sessions. Curr. Biol. **28**, 3661-3666. (10.1016/j.cub.2018.09.065) 30416063

[13] Schulz TM, Whitehead H, Gero S, Rendell L. 2008 Overlapping and matching of codas in vocal interactions between sperm whales: insights into communication function. Anim. Behav. **76**, 1977-1988. (10.1016/j.anbehav.2008.07.032)

[14] Takahashi DY, Narayanan DZ, Ghazanfar AA. 2013 Coupled oscillator dynamics of vocal turn-taking in monkeys. Curr. Biol. **23**, 2162-2168. (10.1016/j.cub.2013.09.005) 24139740

[15] Yoshida S, Okanoya K. 2005 Evolution of turn-taking: a bio-cognitive perspective. Cogn. Stud. Bullet. Jpn Cogn. Sci. Soc. **12**, 153-165. (10.11225/jcss.12.153)

[16] Henry MJ, Cook PF, de Reus K, Nityananda V, Rouse AA, Kotz SA. 2021 An ecological approach to measuring synchronization abilities across the animal kingdom. Phil. Trans. R. Soc. B **376**, 20200336. (10.1098/rstb.2020.0336) 34420382 PMC8380968

[17] Gamba M, Torti V, Estienne V, Randrianarison RM, Valente D, Rovara P, Bonadonna G, Friard O, Giacoma C. 2016 The Indris have got rhythm! Timing and pitch variation of a primate song examined between sexes and age classes. Front. Neurosci. **10**, 249. (10.3389/fnins.2016.00249) 27378834 PMC4908265

[18] Clink DJ, Tasirin JS, Klinck H. 2020 Vocal individuality and rhythm in male and female duet contributions of a nonhuman primate. Curr. Zool. **66**, 173-186. (10.1093/cz/zoz035) 32440276 PMC7233616

[19] Fuhrmann D, Ravignani A, Marshall-Pescini S, Whiten A. 2014 Synchrony and motor mimicking in chimpanzee observational learning. Sci. Rep. **4**, 1-7. (10.1038/srep05283) PMC5381545 24923651

[20] Ravignani A. 2019 Timing of antisynchronous calling: a case study in a harbour seal pup (*Phoca vitulina*). J. Comp. Psychol. **133**, 272-277. (10.1037/com0000160) 30550302

[21] Takahashi DY, Fenley AR, Ghazanfar AA. 2016 Early development of turn-taking with parents shapes vocal acoustics in infant marmoset monkeys. Phil. Trans. R. Soc. B **371**, 20150370. (10.1098/rstb.2015.0370) 27069047 PMC4843608

[22] Roeske TC, Tchernichovski O, Poeppel D, Jacoby N. 2020 Categorical rhythms are shared between songbirds and humans. Curr. Biol. **30**, 3544-3555. (10.1016/j.cub.2020.06.072) 32707062 PMC7511425

[23] De Gregorio C, Valente D, Raimondi T, Torti V, Miaretsoa L, Friard O, Giacoma C, Ravignani A, Gamba M. 2021 Categorical rhythms in a singing primate. Curr. Biol. **31**, R1379-R1380. (10.1016/j.cub.2021.09.032) 34699799

[24] van der Steen MC, Keller PE. 2013 The ADaptation and Anticipation Model (ADAM) of sensorimotor synchronization. Front. Hum. Neurosci. **7**, 253. (10.3389/fnhum.2013.00253) 23772211 PMC3677131

[25] van der Steen MC, Jacoby N, Fairhurst MT, Keller PE. 2015 Sensorimotor synchronization with tempo-changing auditory sequences: modeling temporal adaptation and anticipation. Brain Res. **1626**, 66-87. (10.1016/j.brainres.2015.01.053) 25725379

[26] Mills PF, Harry B, Stevens CJ, Knoblich G, Keller PE. 2019 Intentionality of a co-actor influences sensorimotor synchronisation with a virtual partner. Q. J. Exp. Psychol. (Colchester) **72**, 1478-1492. (10.1177/1747021818796183) 30081732

[27] Ravignani A, Kello CT, de Reus K, Kotz SA, Dalla Bella S, Méndez-Aróstegui M, Rapado-Tamarit B, Rubio-Garcia A, de Boer B. 2019 Ontogeny of vocal rhythms in harbour seal pups: an exploratory study. Curr. Zool. **65**, 107-120. (10.1093/cz/zoy055) 30697246 PMC6347067

[28] Patel AD. 2006 Musical rhythm, linguistic rhythm, and human evolution. Music Percept. **24**, 99-104.

[29] Patel AD. 2014 The evolutionary biology of musical rhythm: was Darwin wrong? PLoS Biol. **12**, 1001821. (10.1371/journal.pbio.1001821) PMC3965380 24667562

[30] Keehn RJJ, Iversen JR, Schulz I, Patel AD. 2019 Spontaneity and diversity of movement to music are not uniquely human. Curr. Biol. **29**, R621-R622. (10.1016/j.cub.2019.05.035) 31287976

[31] Schusterman RJ. 1977 Temporal patterning in sea lion barking (*Zalophus californianus*). Behav. Biol. **20**, 404-408. (10.1016/S0091-6773(77)90964-6)

[32] Ralls K, Fiorelli P, Gish S. 1985 Vocalizations and vocal mimicry in captive harbor seals, *Phoca vitulina*. Can. J. Zool. **63**, 1050-1056. (10.1139/z85-157)

[33] Cook P, Rouse A, Wilson M, Reichmuth C. 2013 A California sea lion (*Zalophus californianus*) can keep the beat: motor entrainment to rhythmic auditory stimuli in a non vocal mimic. J. Comp. Psychol. **127**, 412-427. (10.1037/a0032345) 23544769

[34] Torres Borda L, Jadoul Y, Rasilo H, Salazar Casals A, Ravignani A. 2021 Vocal plasticity in harbour seal pups. Phil.Trans. R. Soc. B **376**, 20200456. (10.1098/rstb.2020.0456) 34719248 PMC8558775

[35] Sauvé CC, Beauplet G, Hammill MO, Charrier I. 2015 Acoustic analysis of airborne, underwater, and amphibious mother attraction calls by wild harbor seal pups (*Phoca vitulina*). J. Mammal. **96**, 591-602. (10.1093/jmammal/gyv064)

[36] Perry EA, Renouf D. 1988 Further-studies of the role of harbor seal (*Phoca vitulina*) pup vocalizations in preventing separation of mother pup pairs. Can. J. Zool. **66**, 934-938. (10.1139/z88-138)

[37] Khan CB, Markowitz H, McCowan B. 2006 Vocal development in captive harbor seal pups, *Phoca vitulina richardii*: age, sex, and individual differences. J. Acoust. Soc. Am. **120**, 1684-1694. (10.1121/1.2226530) 17004489

[38] Chow CP, Mitchell JF, Miller CT. 2015 Vocal turn-taking in a non-human primate is learned during ontogeny. Proc. R. Soc. B **282**, 20150069. (10.1098/rspb.2015.0069) PMC4424641 25904663

[39] Landler L, Ruxton GD, Malkemper EP. 2018 Circular data in biology: advice for effectively implementing statistical procedures. Behav. Ecol. Sociobiol. **72**, 1-10. (10.1007/s00265-018-2538-y) PMC6060829 30100666

[40] Ruxton GD. 2017 Testing for departure from uniformity and estimating mean direction for circular data. Biol. Lett. **13**, 20160756. (10.1098/rsbl.2016.0756) 28100719 PMC5310581

[41] Pewsey A, Neuhäuser M, Ruxton GD. 2013 Circular statistics in R. Oxford, UK: Oxford University Press.

[42] Zar JH. 2010 Circular distributions: hypothesis testing. In Biostatistical analysis (ed. JH Zar), pp. 624-668. Upper Saddle River, NJ: Pearson Prentice Hall.

[43] Landler L, Ruxton GD, Malkemper EP. 2021 Advice on comparing two independent samples of circular data in biology. Sci. Rep. **11**, 1-10. (10.1038/s41598-021-99299-5) 34645855 PMC8514454

[44] Granger CWJ. 1969 Investigating causal relations by econometric models and cross-spectral methods. Econometrica **37**, 424-438. (10.2307/1912791)

[45] Ravignani A, Norton P. 2017 Measuring rhythmic complexity: a primer to quantify and compare temporal structure in speech, movement, and animal vocalizations. J. Lang. Evol. **2**, 4-19. (10.1093/jole/lzx002)

[46] Zeileis A, Hothorn T. 2002 Diagnostic checking in regression relationships. R News **2**, 7-10. (https://CRAN.R-project.org/doc/Rnews/)

[47] Keller PE, Novembre G, Hove MJ. 2014 Rhythm in joint action: psychological and neurophysiological mechanisms for real-time interpersonal coordination. Phil. Trans. R. Soc. Lond. B **369**, 20130394. (10.1098/rstb.2013.0394) 25385772 PMC4240961

[48] Keller PE, Novembre G, Loehr J. 2016 Musical ensemble performance: representing self, other and joint action outcomes. In Shared representations: sensorimotor foundations of social life (eds SS Obhi, ES Cross), pp. 280-310. Cambridge, UK: Cambridge University Press.

[49] Mills PF, van der Steen MC, Schultz BG, Keller PE. 2015 Individual differences in temporal anticipation and adaptation during sensorimotor synchronization. Timing Time Percept. **3**, 13-31. (10.1163/22134468-03002040)

[50] van der Steen MC, Schwartze M, Kotz SA, Keller PE. 2015 Modeling effects of cerebellar and basal ganglia lesions on adaptation and anticipation during sensorimotor synchronization. Ann. N Y Acad. Sci. **1337**, 101-110. (10.1111/nyas.12628) 25773623

[51] Lemasson A, Pereira H, Levréro F. 2018 Social basis of vocal interactions in western lowland gorillas (*Gorilla g. gorilla*). J. Comp. Psychol. **132**, 141-151. (10.1037/com0000105) 29528666

[52] Yang XJ, Ma XR, Slabbekoorn H. 2014 Timing vocal behaviour: experimental evidence for song overlap avoidance in Eurasian wrens. Behav. Processes **103**, 84-90. (10.1016/j.beproc.2013.11.011) 24309317

[53] Song J, Sun R, Fang K, Zhang B, Fang G. 2020 Flexibility as a strategy for avoiding call overlap in male Anhui treefrogs. Asian Herpetol. Res. **11**, 230-239. (10.16373/j.cnki.ahr.1900331)

[54] Serrano A, Terhune JM. 2002 Antimasking aspects of harp seal (*Pagophilus groenlandicus*) underwater vocalizations. J. Acoust. Soc. Am. **112**, 3083-3090. (10.1121/1.1518987) 12509031

[55] Verga L, Sroka MG, Varola M, Villanueva S, Ravignani A. 2022 Spontaneous rhythm discrimination in a mammalian vocal learner. Biol. Lett. **18**, 20220316. 36285461 10.1098/rsbl.2022.0316 PMC9597408

[56] Deecke VB, Slater PJ, Ford JK. 2002 Selective habituation shapes acoustic predator recognition in harbour seals. Nature **6912**, 171-173. (10.1038/nature01030) 12432391

[57] Bigelow AE, DeCoste C. 2003 Sensitivity to social contingency from mothers and strangers in 2-, 4-, and 6-month-old infants. Infancy **4**, 111-140. (10.1207/S15327078IN0401_6)

[58] Henning A, Striano T. 2011 Infant and maternal sensitivity to interpersonal timing. Child Dev. **82**, 916-931. (10.1111/j.1467-8624.2010.01574.x) 21410930

[59] Malloch S, Trevarthen C. 2018 The human nature of music. Front. Psychol. **9**, 1680. (10.3389/fpsyg.2018.01680) 30337892 PMC6180173

[60] Soussignan R, Nadel J, Canet P, Gerardin P. 2006 Sensitivity to social contingency and positive emotion in 2-month-olds. Infancy **10**, 123-144. (10.1207/s15327078in1002_2)

[61] Stormark KM, Braarud HC. 2004 Infants' sensitivity to social contingency: a ‘double video’ study of face-to-face communication between 2- and 4-month-olds and their mothers. Infant Behav. Dev. **27**, 195-203. (10.1016/j.infbeh.2003.09.004)

[62] Fairhurst MT, Janata P, Keller PE. 2013 Being and feeling in sync with an adaptive virtual partner: brain mechanisms underlying dynamic cooperativity. Cereb. Cortex **23**, 2592-2600. (10.1093/cercor/bhs243) 22892422

[63] Fairhurst MT, Janata P, Keller PE. 2014 Leading the follower: an fMRI investigation of dynamic cooperativity and leader-follower strategies in synchronization with an adaptive virtual partner. Neuroimage **84**, 688-697. (10.1016/j.neuroimage.2013.09.027) 24064075

[64] Repp BH, Keller PE. 2008 Sensorimotor synchronization with adaptively timed sequences. Hum. Mov. Sci. **27**, 423-456. (10.1016/j.humov.2008.02.016) 18405989

[65] Harry B, Keller PE. 2019 Tutorial and simulations with ADAM: an adaptation and anticipation model of sensorimotor synchronization. Biol. Cybern. **113**, 397-421. (10.1007/s00422-019-00798-6) 30963226

[66] Patel AD, Iversen JR, Bregman MR, Schulz I. 2009 Experimental evidence for synchronization to a musical beat in a nonhuman animal. Curr. Biol. **19**, 827-830. (10.1016/j.cub.2009.03.038) 19409790

[67] Anichini M, de Reus K, Hersh TA, Valente D, Salazar-Casals A, Berry C, Keller PE, Ravignani A. 2023 Measuring rhythms of vocal interactions: a proof of principle in harbour seal pups. Figshare. (10.6084/m9.figshare.c.6412280) PMC9985970 36871583

